# Evaluation of effect of cooled haemodialysis on cognition in patients with end-stage kidney disease (ECHECKED) feasibility randomised controlled trial results

**DOI:** 10.1186/s12882-024-03883-6

**Published:** 2024-12-19

**Authors:** Indranil Dasgupta, Aghogho Odudu, Jyoti Baharani, Niall Fergusson, Helen Griffiths, John Harrison, Awais Hameed, Paul Maruff, Louise Ryan, Neil Thomas, Gavin Woodhall, George Tadros

**Affiliations:** 1 Renal Unit, Heartlands Hospital, Bordesley Green East, Birmingham, B9 5SS UK; 2 Warwick Medical School, University of Warwick, Coventry, CV4 7AL UK; 3 Division of Cardiovascular Sciences, University of Manchester, Manchester, M13 9PL UK; 4 Manchester University NHS Foundation Trust, Manchester, M13 9PWL UK; 5 Department of Care of the Elderly, Heartlands Hospital, Birmingham, B9 5SS UK; 6 Faculty of Medicine, Health and Life Science, Swansea University, Swansea, SA1 8EN UK; 7 Institute of Psychiatry, Psychology & Neuroscience, King’s College London, London, WC2R 2LS UK; 8 Cogstate Limited, Melbourne, VIC 3000 Australia; 9 Institute of Applied Health Research, University of Birmingham, Birmingham, B15 2SQ UK; 10 School of Neuropharmacology, Aston University, Birmingham, B4 7ET UK; 11 Department of Old Age Psychiatry, Heartlands Hospital, Birmingham, B9 5SS UK; 12 Aston Medical School, Aston University, Birmingham, B4 7ET UK

**Keywords:** Cognition, Cooled dialysate, Feasibility trial, Haemodialysis, Randomised controlled trial

## Abstract

**Background:**

Cognitive impairment is common in haemodialysis patients with no known beneficial interventions. Cooler dialysate slows brain white-matter changes, but its effect on cognition is unknown. This feasibility trial was performed to inform a fully-powered, randomised trial to assess this.

**Methods:**

We aimed to randomise (1:1) 90 haemodialysis patients to this double-blinded, randomised controlled feasibility trial to standard care (dialysate-temperature 36.5 °C) or intervention (35 °C). Eligible patients were adult chronic haemodialysis recipients with no established diagnosis of dementia or psychiatric disease. The primary outcome was change in Montreal Cognitive Assessment (MoCA) score at 12-months. Secondary outcomes included recruitment and attrition rates, reasons for non-recruitment, intradialytic hypotension, depression, patient burden, computerised cognition test battery, and quality of life.

**Findings:**

Of 334 patients screened, 160 were eligible. 99 declined mainly for the extra non-dialysis day study visits. Sixty-one patients consented, 43 randomised – 20 in standard care, 23 in intervention arms; 13 withdrew for non-dialysis day visits and 5 without reason before randomisation. 27 patients (12 standard care, 15 intervention) completed the trial – 5 died, 1 transplanted, 4 withdrew consent, and 6 could not attend due to the pandemic. Low temperature dialysis was well tolerated. There was no difference in change in MoCA from baseline to 12 months between the standard and intervention arms; 1.0 (-2.8–3.0, *p* = 0.755) and − 2.0 (-1.0 - -4.0, *p* = 0.047) respectively. There were no differences between groups on any secondary measures. There were no significant adverse events reported.

**Discussion:**

The trial was significantly affected by the COVID-19 pandemic contributing to an attrition rate of 27%. The non-dialysis day research visits were mainly responsible for low recruitment and consent withdrawal. There are several learning points, described in the article, which will inform design of definitive trials in this area in the future.

**Trial registration:**

ClinicalTrials.gov Identifier NCT03645733. Registration date 24/08/2018.

**Supplementary Information:**

The online version contains supplementary material available at 10.1186/s12882-024-03883-6.

## Introduction

The prevalence of cognitive impairment (CI) increases with progression of chronic kidney disease (CKD) [1, 2]. Moderate to severe CI is present in 30–70% of haemodialysis (HD) patients [3–5]. Several HD factors have been implicated including oxidative stress, malnutrition and inflammation, uraemic neurotoxins, and intradialytic hypotension (IDH) [6–9]. Although the brain has auto regulation, which protects it against a wide range of abnormal blood pressures, the lower level of cerebral autoregulation varies greatly in patients receiving haemodialysis [10]. The cerebral arterial mean velocity flow declines significantly during dialysis, and this correlates significantly with a decrease in cognitive function [11]. IDH affects patients during 30–40% of HD treatments and leads to repeated ischemic insults to organs including the brain [12–14], which may manifest as altered cognition. Therefore, preventing IDH might plausibly prevent intradialytic brain ischemia and slow the development of CI [15]. Indeed, extended overnight HD, by allowing slower fluid removal, has been shown to improve cognition in a small study [16]. Cooler dialysate (34–35 °C) reduces IDH compared to standard temperature dialysis by 70% [17, 18], by preventing systemic vasodilation, but is underused for perceived thermal symptoms [18–21]. A small clinical trial of cooled dialysate showed stabilisation of white matter on MRI compared to the control group through amelioration of haemodynamic instability [22, 23]. These results suggest there is potential for dialysate cooling to operate as a neuroprotective treatment. The low usage of cooler dialysate in the United Kingdom affords an opportunity to test this simple, no-cost modification to HD as a potential intervention to prevent CI. Before this hypothesis can be challenged there remain uncertainties around the study design of a definitive trial of cooled dialysate and CI for example there is little information about how well cooled HD is tolerated. Therefore, we performed this feasibility trial to inform the development of a fully-powered, randomised, controlled trial (RCT) that would examine the efficacy of cooler dialysate in reducing cognitive decline in patients receiving HD for End-Stage Kidney Disease (ESKD).

## Materials and methods

The detailed methodology of this trial is published elsewhere [24]. In brief, this was a multi-site, prospective, randomised, double-blinded, controlled, feasibility trial [25] and adhered to the CONSORT guideline. Patient involvement was at the heart of this study with a service user representative contributing to the design of the study and leading on decisions regarding assessment frequency, timing and setting [24]. Additionally, service users advised on patient information sheets and helped write plan English summaries.

### Participants

Inclusion criteria: Patients aged ≥ 18 years, receiving HD three times a week for ESKD for ≥ 3 months and having mental capacity to give informed consent. While the inclusion criteria required participants to speak English, the assessments were also available in Urdu and Bengali to increase the inclusion of people from ethnic minorities in the study [24]. These two languages were chosen as a local audit identified them as the two most common non-English languages spoken by the study population. Exclusion criteria: Patients with an established diagnosis of dementia or psychiatric condition; receiving cognition altering drugs; inter-current infection; awaiting living donor kidney transplantation within 12 months; expected to survive < 12 months; patients prone to IDH or cardiovascular instability during HD as they were already receiving cooled dialysis haemodialysis (at 36 C) or had reason not to be and those currently involved in another intervention trial.

### Dialysis setting

The trial was conducted in 4 satellite haemodialysis units under the Renal Unit of Heartlands Hospital Birmingham which dialyse established and clinically stable patients with ESKD. Patients set up their own dialysis as much as possible based on their dexterity and competence and were encouraged to be more involved in their care during the pandemic due to staff shortages. The ambient temperature was maintained between 18 C and 24 C depending on the time of year. The standard dialysate flow and blood flow rate are 500 ml/min and 400 ml/min respectively. 70% of the patients are dialysed in a chair with the remainder in a bed. No exercise or meals occur during dialysis but they are offered tea and biscuits. The Montreal Cognitive Assessment (MoCA) test was done on paper and the Cogstate on a laptop.

### Study intervention

After a two-weeks run-in phase to establish patients’ pre-dialysis temperature (with tympanic thermometer), the intervention group started with dialysate temperature of 36 °C which was reduced fortnightly by 0.5 °C until a temperature of 35 °C was reached. Both patients and investigators were blinded to group allocation, the clinical nursing staff was unblinded and temperature display on the machine was concealed. If a lower temperature was not tolerated, the temperature increased back to the previous setting [24].

### Primary outcome measure

The main outcome for this study was differences between the standard temperature (ST) and low temperature (LT) groups in change in cognitive function (MoCA score) from the baseline to 12-month. This endpoint was chosen to inform power and sample size calculations for the definitive RCT and ensure its feasibility.

### Secondary outcome measures

Frequency of IDH along with interdialytic weight gain, ultrafiltration (UF) volume and UF rate as an explanatory outcome, recruitment and attrition rates, non-recruitment reasons, depression and anxiety rates, acceptability, and usability of Cogstate with composite cognitive score as an outcome, quality of life (QOL), activities of daily living (ADL) and carers’ burden.

### Data collection

Outcomes were measured at baseline (defined as start of trial rather than initiation of haemodialysis), 6 and 12 months by a blinded rater, on a non-dialysis day, when the best performance on cognitive testing was expected [26]. Supplemental Table 1 summarises the schedule of events.

Patients’ tolerability of low temperature was assessed, using a locally designed and internally validated questionnaire (Supplemental Box 1). This questionnaire was developed and optimised with the help of patients receiving HD at the University Hospitals Birmingham. Adherence to the allocated dialysate temperature was regularly monitored. Delirium was excluded by the Confusion Assessment method (CAM) [27].

The Montreal Cognitive Assessment (MoCA) [28] was the primary outcome measure. MoCA was adjusted for education level. Cognition was also measured using the Cogstate battery, a validated and brief (20–30 min), portable and language-independent computerised test battery [29]. The Cogstate battery was chosen as it has been shown to detect clinically important improvements and decline in cognition associated with a variety of central nervous system disorders and is readily administered by non-experts. Furthermore, performance on the Cogstate tests is strongly correlated with paper tests such as the Trail Making and Digit Symbol Substitution tests. Cogstate provides multiple alternative forms to diminish the practice effects that can occur when conventional tests are readministered to non-demented adults.

Cognitive impairment is a known determinant of QOL in advanced kidney disease [30] and has been demonstrated to affect ADL [31]. As such we measured QOL by Assessment of Quality of Life (AQoL-6D) scale [32] and ADL by The Bristol Activity of Daily Living Scale (BADLS) [33]. As patients receiving haemodialysis who suffer with depressive symptoms perform worse on cognitive testing [34], depression was measured by Hospital Anxiety and Depression Scale (HADS) [35, 36]. The carer burden was assessed by Caregiver Burden scale (CBS) [37].

BP measurement every 30 min during HD was planned. Measurements were taken with a validated automated machine (Welch Allyn or Datascope Accurator). Standard measurements in the units are pre and post HD and one during dialysis, with additional if indication present. IDH was defined as a fall in systolic BP > 20% from baseline during HD, absolute systolic BP < 90mmHg or symptomatic IDH needing intervention.

Interdialytic weight gain, ultrafiltration volume and ultrafiltration rate were extracted from clinical records. The HD recovery time [38] was assessed by, “How long does it take you to recover from a dialysis session?’’.

### Sample size

At least 30 patients in each arm were computed as necessary to identify sample variability that would enable computations of statistical power for hypotheses testing in a definitive study [39]. With 45 patients in each arm, and if the mean (SD) value of the MoCA is 27(2) in the control and intervention arms at the study start, we could expect a 95% confidence interval to range from 26.4 to 27.6 in each arm, giving adequate precision for the estimate required in the study.

### Randomisation

Participants were randomised on a 1:1 basis to the control group (36.5 °C) or cooled dialysate group (35 °C), using Sealed Envelope’s (London, UK) randomisation software. Randomisation was stratified by age group (patients under 55 years of age, 55–75 and above 75).

### Data analysis

Mixed method analysis was planned. The qualitative component, using thematic analysis on semi-structured interviews of patients and carers, was cancelled due to poor recruitment of carers and a reluctance to extend the assessment time for patients when non-dialysis day visits were proving to be a deterrent to recruitment and retention. The aim of the qualitative component was to assess issues related to patient recruitment, practicalities of implementing cooler dialysate, adherence to treatment and effectiveness of the blinding process. The quantitative analysis methods are described below.

### Statistical methods

Data were recorded and analysed using IBM SPSS Statistics for Windows 27.0 (IBM Corp., Armonk, N.Y., USA). Normally distributed data are presented as mean and standard deviation (SD) and non-normal data as median and inter-quartile range (IQR). Related-samples Wilcoxon signed rank test was used to compare median of differences from baseline to 12 months. Independent samples median test was used to compare the difference between two treatment arms at 12 months. Statistical significance was determined using p-value with values of < 0.05 classified as being significant.

For each of the seven test variables of Cogstate, difference in score from baseline to 12 months was calculated and standardised transformation performed. A composite score, using an average standardised score, was also computed for each participant. Difference in mean in the two groups at 12 months were analysed using the independent samples t-tests. A repeated measures ANOVA was used for MoCA at baseline and 12 months. Study participants with missing data were excluded from analysis.

### Ethics and governance

The trial was approved by National Research Ethics Service Committee, West Midlands-South Birmingham (IRAS ID 234107). The study was performed in accordance with the Research Governance Framework, International Conference on Harmonisation Good Clinical Practice Guideline and the Declaration of Helsinki. The study was audited and monitored by University Hospitals Birmingham NHS Foundation Trust as the Study Sponsor.

## Results

**Fig. 1:**
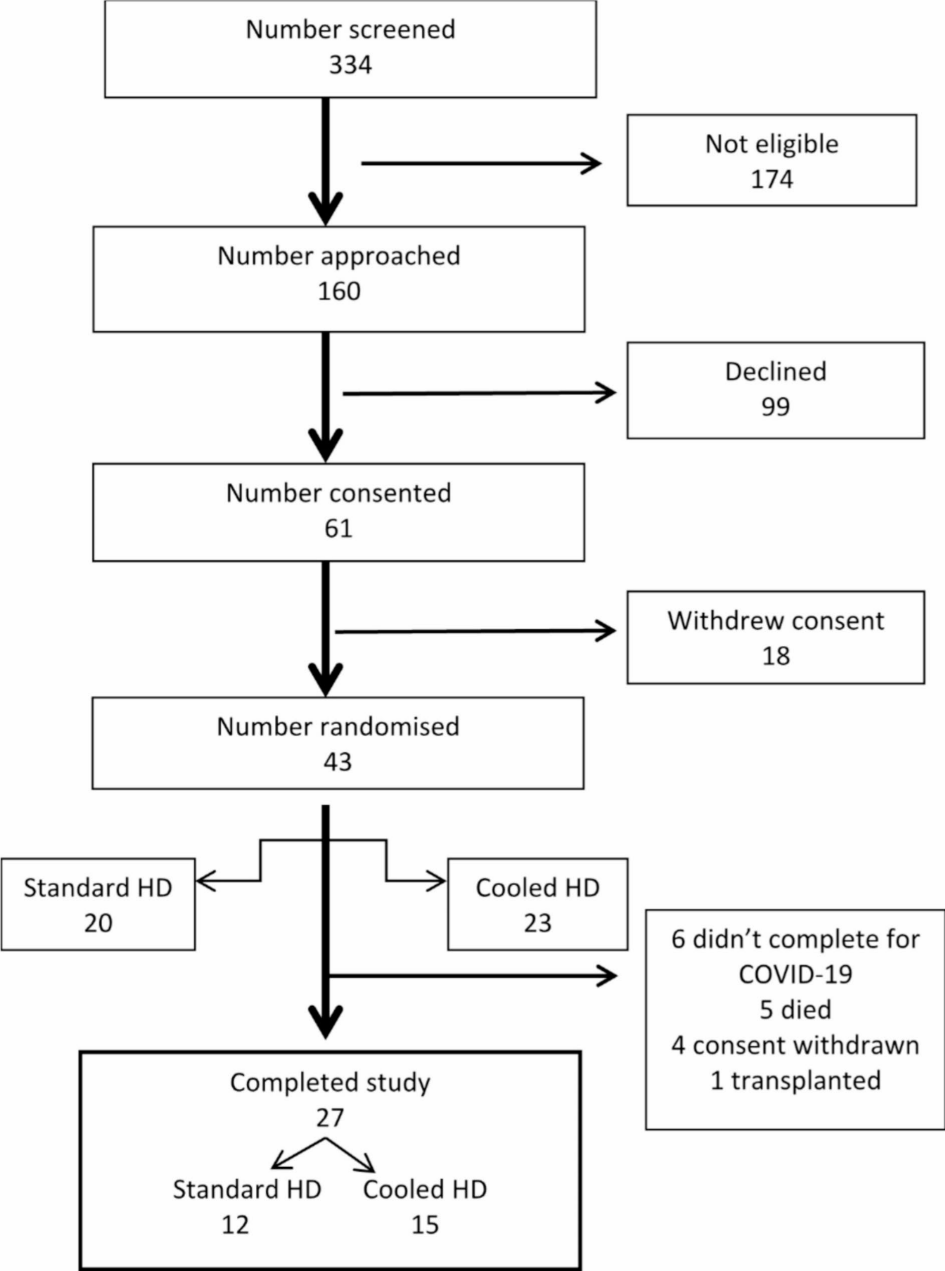
Patient flow in ECHECKED Trial

Of 334 HD patients screened, 174 patients were ineligible, most commonly for limited fluent English language skills, (48) or already receiving cooled dialysate (47). Out of 334 patients, 160 were invited to participate, of which 99 declined. Sixty-one patients consented, of whom 43 were randomised (Fig. 1). Of the 43 patients randomised, 27 completed 12-month follow-up − 12 in ST and 15 in LT arms and were included in the per-protocol analyses. The baseline characteristics of the 43 participants who were randomised are shown in Table 1 while the characteristics of the 27 participants who completed 12-month follow-up are shown in Supplemental Table 2. Recruitment began in January 2018 and follow up ended in November 2020.

**Table 1:** Baseline demographic and clinical parameters of the study population

	Standard	Low	Total
*N*	20	23	43
Age (years)	67 (55–76)	68 (59–77)	68 (59–77)
**Gender**			
Male	13 (65)	16 (70)	29 (67)
Female	7 (35)	7 (30)	14 (33)
**Ethnicity**			
Caucasian	14 (70)	13 (56)	27 (63)
Asian	1 (5)	5 (22)	6 (14)
African	5 (25)	5 (22)	10 (23)
Time on Dialysis (months)	60 (37–99)	56 (37–118)	58 (37–114)
MOCA	21.9 ± 4.6	20.7 ± 6.2	21.2 ± 5.5
HADS Depression	6.5 ± 3.5	4.8 ± 2.4	5.6 ± 3.0
HADS Anxiety	3.0 (1.0–8.0)	3.0 (1.0–4.0)	3.0 (1.0–5.5)
AQOL	48.1 ± 9.6	45.6 ± 8.2	47.7 ± 8.8
PreHD SBP (mmHg)	147.2 ± 24.1	140.0 ± 20.4	143.3 ± 22.2
preHD DBP (mmHg)	72.9 ± 13.4	65.9 ± 10.3	69.0 ± 12.2
Post HD SBP (mmHg)	130.9 ± 21.0	132.6 ± 14.3	131.9 ± 17.4
Post HD DBP (mmHg)	62.0 ± 12.5	63.9 ± 10.5	63.0 ± 11.3
Kt/V	1.33 ± 0.16	1.36 ± 0.26	1.34 ± 0.22
Hb (g/dL)	11.5 ± 0.94	11.0 ± 1.17	11.2 ± 1.09
Alb (g/L)	32.8 ± 3.9	31.4 ± 4.2	32.1 ± 4.1
C.Ca (mmol/L)	2.40 ± 0.15	2.40 ± 0.12	2.40 ± 0.13
Phosphate (mmol/L)	1.60 (1.41–2.04)	1.30 (1.18–1.70)	1.50 (1.19–1.84)
PTH (pmol/L)	48.2 ± 28.0	45.5 ± 23.9	46.7 ± 25.6

Data presented as either Mean ± SD; Median (IQR); *N* (%)

### Primary outcome

The median MoCA score at baseline was 22.5 (IQR: 17.8–25.8) and 25 (IQR: 17.0–26.0) in ST and LT arms respectively. At the 12-month assessment, MoCA scores were 23.5 (16.5–27.0) and 21.0 (15.0–25.0) respectively. The mean change from baseline to 12 months in ST arm was 1.0 (-2.8–3.0, *p* = 0.755) and in LT arm was − 2.0 (-1.0 - -4.0, *p* = 0.047) with no difference between the arms in change in MoCA score at 12 months (*p* = 0.449, Table 2), or on repeated measures analysis. There was also no statistically significant change in the individual domains of MoCA from baseline to 12 months.

**Table 2:** Primary and main secondary outcomes at 12 months

	Standard	Low	Difference in change (*p* =)
**MOCA**			
*N*	**12**	**15**	
Baseline	22.5 (17.8–25.8)	25.0 (17.0–26.0)	
12 months	23.5 (16.5–27.0)	21.0 (15.0–25.0)	0.449
Change from baseline, p	1.0 (-2.8–3.0) *p* = 0.755	2.0 (-1.0–4.0) *p* = 0.047	
**HADS Depression**			
*N*	12	15	
Baseline	4.5 (3.0–6.8)	4.0 (3.0–7.0)	
12 months	5.0 (3.3–5.0)	5.0 (4.0–7.0)	0.236
Change from baseline, p	-0.5 (-2.0–2.8) *p* = 0.521	0.0 (-3.0–2.0) *p* = 0.503	
**HADS Anxiety**			
*N*	12	15	
Baseline	2.5 (0.3–6.5)	3.0 (1.0–4.0)	
12 months	2.5 (1.0–6.0)	4.0 (1.0–7.0)	0.704
Change from baseline, p	-0.5 (-1.0–1.8) *p* = 0.834	0.0 (-3.0–1.0) *p* = 0.194	
**AQOL**			
*N*	**9**	**12**	
Baseline	47.0 (33.5–52.0)	46.0 (41.5–51.5)	
12 months	47.0 (41.5–55.0)	47.5 (37.5–52.8)	0.66
Change from baseline, p	-1.0 (-10.0–2.0) *p* = 0.326	-1.0 (-9.5–8.0) *p* = 0.859	
**HD Recovery time**			
*N*	9	11	
Baseline (hours)	4 (2–18)	3 (2–6)	
12 months (hours)	8 (3–12)	4 (2–8)	0.67
Change from baseline (hours), p	0 (-8–2) *p* = 0.397	0 (-1–4) *p* = 0.813	

Data presented as median (IQR)

MoCA – Montreal Cognitive Assessment tool, CAM – Confusion Assessment Method, QoL – quality of life, ADL – activities of daily living, HADS – Hospital Anxiety and Depression Scale, HD – haemodialysis

### Secondary outcomes

There were 39 recorded IDH episodes in 12 months in LT (median 0, range 0–19) and 23 in ST arms (median 1, range 0–5, *p* = 0.60). Interdialytic weight gain, UF volume and UF rate were inconsistently recorded.

The recruitment rate is shown in Fig. 1. Of the 160 patients who were approached to participate, 31 (19.4%) refused to be randomised to LT arm and 68 (42.5%) were unhappy to attend additional appointments on a non-dialysis day.

Eighteen patients withdrew consent before randomisation – 5 did not give a reason, 13 for the extra visit on a non-dialysis day. Of the 43 patients randomised, 63% completed the study; 5 patients died, 1 received a kidney transplant, 4 refused to attend and 6 could not attend the final visit because of the pandemic, giving an attrition rate of 37%.

Six patients in ST (31.5%) and 8 patients in LT arms (34.7%) had a HADS depression score of ≥ 8 at baseline suggesting depression. There was no difference between the groups at baseline or in change from baseline to 12 months in HADS depression and anxiety scores (Table 2). None were confused at baseline as assessed by CAM.

Thirty-five and twenty-seven patients completed the Cogstate battery at 6 and 12 months respectively. There was no difference in change in total z-score or components between the two groups from baseline to 12 months (Table 3).

**Table 3:** Change in Cogstate scores at 6 and 12 months

		Low			Standard			*p*	Mean Difference	Confidence interval	
		*N*	Mean	S.D.	*N*	Mean	S.D.			Lower	Upper
At 6 month											
Detection speed (spd_ZS6)	Psychomotor function	16	-0.04	1.24	15	0.22	0.83	0.499	-0.26	-1.04	0.52
Groton Maze Learning Test (err_ZS6)	Executive function	12	0.07	0.92	11	-0.15	1.23	0.618	0.23	-0.71	1.16
Identification Test (spd_ZS6)	Attention	16	-0.04	1.13	14	0.16	1.00	0.617	-0.20	-1.00	0.60
International Shopping List (wrds_ZS6)	Verbal episodic memory	16	0.12	1.11	15	-0.21	0.91	0.378	0.33	-0.42	1.08
International Shopping List-Delayed recall (wrds_ZS6)	Verbal episodic memory	16	0.16	1.17	15	-0.31	0.84	0.220	0.46	-0.29	1.22
One Back Test (acc_ZS6)	Working memory-accuracy	14	0.22	1.16	15	0.03	0.91	0.634	0.19	-0.61	0.98
One Back Test (spd_ZS6)	Working memory-speed	14	-0.18	1.07	15	0.22	0.84	0.272	-0.40	-1.13	0.33
Avg ZS6		17	0.06	0.53	15	0.00	0.27	0.674	0.06	-0.25	0.37
At 12 month											
Detection Speed (spd_ZS12)	Psychomotor function	16	-0.23	0.98	5	0.80	0.73	0.043	-1.03	-2.03	-0.04
Groton Maze Learning Test (err_ZS12)	Executive function	14	-0.01	1.16	5	0.08	0.78	0.867	-0.10	-1.29	1.09
Identification Test (spd_ZS12)	Attention	16	-0.33	1.04	5	1.02	0.35	0.011	-1.35	-2.36	-0.35
International shopping list (wrds_ZS12)	Verbal episodic memory	16	-0.19	1.04	5	0.52	1.09	0.202	-0.71	-1.83	0.41
International shopping list-Delayed recall (wrds_ZS12)	Verbal episodic memory	16	-0.23	0.94	6	0.17	1.11	0.409	-0.40	-1.38	0.58
One back test (acc_ZS12)	Working memory-accuracy	15	0.41	0.75	5	-0.88	1.20	0.010	1.29	0.34	2.23
One back test (spd_ZS12)	Working memory- speed	15	0.08	1.01	5	-0.24	0.78	0.527	0.32	-0.73	1.37
Avg ZS12		17	-0.06	0.42	10	-0.01	0.93	0.836	-0.05	-0.59	0.48

ZS - Z score

Carers’ burden assessment was not analysed as only 4 carers consented.

Assessment of QOL (AQoL-6D) was done in all patients at baseline; 12 in ST and 15 in LT arm had repeated assessment at 12 months. There was no difference in change in AQoL-6D score at 12 months. ADL (BADLS) was measured in 8 patients (2 in ST and 6 in LT arm) at baseline and was not repeated.

All patients had recovery time assessed at baseline; 9 in LT and 11 in ST arm had it repeated at 12 months. There was no difference between the groups in change in recovery time (Table 2).

Tolerability of dialysis temperature was assessed at 2, 4 and 6 weeks in both groups by an internally validated questionnaire (Supplemental Box 1). Compared to standard temperature dialysis, low temperature dialysis was well tolerated (Table 4). There were no significant adverse events reported.

**Table 4:** Tolerability of dialysis between the two treatment arms

	Standard	Low
**Felt Cold (%)**	43	50
**General Discomfort (%)**	24	17
**Numbness and Pain (%)**	43	17
**Sleepier (%)**	50	17
**More tired (%)**	57	17
**Worse Concentration (%)**	24	9

As follow-up was curtailed because of the pandemic, we performed non-specified, secondary analyses for the main outcomes at 6 months. Thirty-five patients completed 6-month follow-up – 17 in ST and 18 in LT arm. There were no differences between groups in change from baseline to 6 months MoCA Score, and Anxiety scores, AQOL, recovery time or composite Cogstate score (Tables 3 and 5). Although there was a difference in the HADS Depression score at 6 months this was not present at 12 months.

**Table 5:** Secondary analysis on main outcomes at 6 months

	Standard	Low	Difference in change (*p* =)
**MOCA**			
*N*	**17**	**18**	
Baseline	22.0 (17.0–25.0)	23.0 (17.8–26.0)	
6 months	24.0 (16.0–25.5)	22.0 (18.0–26.0)	0.241
Change from baseline, p	0.0 (-2.0–2.5) *p* = 0.697	0.0 (-2.0 − 1.3) *p* = 0.588	
**HADS Depression**			
*N*	16	18	
Baseline	6.0 (3.3–9.0)	4.0 (2.8–7.0)	
6 months	3.0 (1.0–7.0)	3.5 (0.8–7.3)	< 0.001
Change from baseline, p	-1.0 (-2.8–0.8) *p* = 0.007	0.5 (-0.3–2.0) *p* = 0.449	
**HADS Anxiety**			
*N*	16	18	
Baseline	3.0 (0.3–8.5)	2.0 (1.0–4.3)	
6 months	5.0 (3.3–10.8)	4.5 (2.8–6.0)	0.774
Change from baseline, p	0.5 (-1.8–2.8) *p* = 0.078	0.5 (-2.0–2.0) *p* = 0.017	
**AQOL**			
*N*	**16**	**18**	
Baseline	48.5 (10.2)	46.2 (8.7)	
6 months	51.1 (10.0)	45.6 (13.2)	0.184
Change from baseline, p	2.6 (5.1) *p* = 0.063	-0.7 (9.2) *p* = 0.763	
**HD Recovery time**			
*N*	15	16	
Baseline (hours)	4 (2–12)	3 (1–12)	
6 months (hours)	6 (2–12)	4 (0–4)	0.821
Change from baseline (hours), p	0 (-4–3) *p* = 1.000	0 (-12–2) *p* = 0.334	

Data presented as Median (IQR); Mean (SD)

MoCA – Montreal Cognitive Assessment tool, CAM – Confusion Assessment Method, QoL – quality of life, ADL – activities of daily living, HADS – Hospital Anxiety and Depression Scale, HD - haemodialysis

## Discussion

This feasibility trial was designed to inform a fully-powered RCT to test the hypothesis that patients treated with conventional HD have lesser cognitive decline and better QoL using cooled dialysate (35 °C) compared to a standard temperature dialysate (36.5 °C). If successful, the treatment could potentially be applied universally at no extra cost.

Of the 334 patients screened, 52% were ineligible. 62% of patients approached declined to participate, the main reason was because of the extra visits required on non-dialysis days. Forty-three patients were randomised − 35 and 27 completed 6- and 12-months follow-up respectively. Leaving the 6 patients who could not attend the final visit due to COVID-19 restrictions, the attrition rate post-randomisation was 27%, slightly higher than the rate expected (20%) when designing the study.

Although there was a decrease in the MoCA over 12 months from baseline in the intervention group (25 to 21, *p* = 0.047) there was no significant difference between treatment and control groups in the primary endpoint for the study, change in MoCA from baseline (Table 2). These results highlight the need for a definitive study.

There were no differences between groups on the secondary endpoints; HADS depression, HADS anxiety, AQOL-6D, recovery time and Cogstate scores. However, this was a feasibility study and aimed to gather information to allow for power calculations for a definitive study rather than be powered to detect a significant difference.

We were unable to assess carers’ burden because of poor recruitment, only 4 of 43 carers consented to participate, and to assess the change in ADL, as only a small number of participants agreed to BDLS assessment because of high question burden.

Conducting clinical trials during the COVID-19 pandemic was challenging [40], especially studies involving HD patients [41]. The pandemic compromised recruitment and follow-up of patients. However, the main issue affecting recruitment was the necessity for participants to attend appointments on non-dialysis days, although they needed to attend only 3 over 12 months. Time commitment and travel requirement have been highlighted by dialysis patients as a barrier to participating in randomised trials [42]. True attrition rate was difficult to estimate due to restricted patient and research staff access during the pandemic.

Maintaining blinding of dialysate temperature to the patient was difficult particularly during the pandemic as many patients transferred units and more set up their own machine. In some cases, symptoms related to low temperature dialysis gave it away. Recording BP every 30 min during HD for 12 months proved challenging as this is not routine practice in the UK. Hence, it was impossible to accurately record the number of asymptomatic IDH episodes, which we planned to test as the explanatory outcome for CI in HD patients.

Reassuringly, 90% of patients were maintained in the allocated dialysate temperature arm despite the disruption to HD services during the pandemic. Contrary to a systematic review which found a fixed reduction in dialysis temperature increased discomfort rates among recipients in our study a fixed reduction in dialysis temperature to 35^o^C was well tolerated when compared to standard temperature dialysis [43].

The main strengths of this study are (1) this is the first trial to assess the effect of cooled dialysate on cognition and QoL in HD patients, (2) the multisite RCT design, and (3) the assessment of a range of outcomes to inform the design of a future large trial.

The main limitations of the study included requirement of a good command of spoken English which excluded many ethnic minority patients, and hence results may not be representative. Secondly, the MoCA is a screening test and may have a low sensitivity to cognitive changes that might occur with cooled dialysate. However, as it is a clinical rating of cognition, any difference observed will provide a reference for the extent to which the cognitive effects have clinical meaningfulness. Thirdly, we were unable to collect qualitative data of patients’ and carers’ experiences of study practicalities and recruitment due to poor recruitment of carers and a reluctance to extend the assessments on non-dialysis days for patients as they were already proving to be a barrier to recruitment. Finally, we did not measure residual kidney function (RKF). HD patients with preserved RKF have previously been shown to have better cognitive function [44]. As IDH can also result in a loss of RKF [45] this could be a potential mediator between IDH and cognitive impairment. This may be further mediated by protein-bound uraemic toxins which have previously been demonstrated to be negatively associated with cognition in people receiving HD [46].

There are several learning points from this pilot study which are important for both designing a fully-powered RCT to evaluate the effect of cooled dialysis on cognition and other future studies examining cognition in haemodialysis patients: (1) Study assessments on a non-dialysis day was the major impediment to recruitment and retention, and hence should be avoided in future trials. (2) Low temperature (35 C) dialysis was well tolerated, and it was possible to maintain patients in their allocated dialysate temperature arm for 12 months suggesting long term tolerability. (3) The study provided an estimate of the variability in the outcome measures to inform power calculation for the future RCT. 4). It is feasible to use Cogstate, a computerised cognitive function test battery in HD patients, for primary outcome assessment in the substantive trial. 5) The use of Cogstate will allow enrolment of patients unable to read or write English in future trials. Additionally, we will allocate funding for translators to allow for inclusion of non-English speaking ethnic minorities. 6) Cogstate has the potential to assess cognitive endpoints remotely, which may reduce study burden and increase statistical power in future studies. 7) BP measurements every 30 min during HD are logistically challenging. 8) Information on interdialytic weight gain, ultrafiltration rate and volume are difficult to collect manually. 9) There was poor uptake in assessments of ALDs and carer’s burden and hence may be removed from the future RCT. 10) Finally, the study highlights the importance of identifying smaller core datasets, minimising the frequency of measurements, and simplifying data collection.

These lessons will be applied in designing the definitive trial assessing the impact of low temperature dialysis on cognition. This will include using a language-neutral cognitive function battery, namely Cogstate, as the primary outcome measure which will allow inclusion of non-English speaking patients, as well as using translators. Cognitive assessment will be incorporated into dialysis visits to aid recruitment and retention of participants, recognising this will need to be performed pre-dialysis or within the first hour of dialysis as there is a transient intradialytic decline in cognitive function towards the later part of a HD session. Service users will be consulted in future study protocol design to ensure acceptability and accessibility, and increase representativeness of a future study population. Continuous BP monitoring during dialysis will be considered to assess the role of asymptomatic hypotension in CI in HD. We will also collect data on residual kidney function in the form of urine output and consider measuring protein-bound toxins to assess if they could be mediators of a potential observed effect of cooled haemodialysis on cognition in the future study. Data on parameters that can affect IDH (UF rate, UF profiling, sodium profiling and use of medications that affect BP such as midodrine, prior or during dialysis, will be collected. Parameters that are routinely measured - e.g. interdialytic weight gain, UF rate and volume, dialysate temperature, biochemical test results, etc. - will be downloaded from HD machines or electronic patient records (EPR). These, the integration of the trial system and the EPR and linkage with the national dialysis registry for outcome data will streamline the process of the future RCT.

## Conclusion

This feasibility trial, conducted to inform the design of a definitive trial investigating the impact of cooled dialysis on CI in HD patients, was disrupted by the COVID-19 pandemic. Furthermore, recruitment and retention of participants was significantly affected by the requirement for the participants to attend non-dialysis day study visits. Despite these, several important lessons were learnt from the study that will help to design definitive trials in this area in the future.

## Electronic supplementary material

Below is the link to the electronic supplementary material.

Supplementary Material 1: Table 1: Schedule of events in ECHECKED trial. Table 2: Baseline characteristics of participants who completed study. Box 1: Questionnaire to assess patient experience of low temperature dialysis.

## Data Availability

The data underlying this article will be shared on reasonable request to the corresponding author.
