# Supplementary material for: Evaluation of effect of cooled haemodialysis on cognition in patients with end-stage kidney disease (ECHECKED) feasibility randomised controlled trial results
Source: BMC Nephrol. 2024 Dec 19;25:466. doi: 10.1186/s12882-024-03883-6 (PMC11657600; doi:10.1186/s12882-024-03883-6)
Supplement: Supplementary file 1 — Supplementary Material 1: Table 1: Schedule of events in ECHECKED trial. Table 2: Baseline characteristics of participants who completed study. Box 1: Questionnaire to assess patient experience of low temperature dialysis. [file 12882_2024_3883_MOESM1_ESM.pdf]

Supplemental Material

Supplemental Table 1: Schedule of events in ECHECKED trial

Supplemental Box 1: Questionnaire to assess patient experience of low temperature dialysis

Table 1: Schedule of events in ECHECKED Trial

	Baseline (O month)	6 months	12 months
Consent	X		
Randomisation	X		
Baseline data (defined)	X		
Cognitive function:<br>Montreal Cognitive Assesment[31]<br>Cogstate[32]<br>Confusion Assessment Method[33]	 X<br>X<br>X	 X<br>X<br>X	 X<br>X<br>X
Tolerability of Low Temperature Dialysis Questionnaire	Every two weeks for the first 6 weeks		
Activities of daily living:<br>Assessment of QoL[34]<br>Bristol Activities of Daily Living Scale[35]	 X<br>X	 X<br>X	 X<br>X
Carer burden assessment[36]	X	X	X
Hospital Anxiety and Depression Scale [37]	X	X	X
HD recovery time[38]	X	X	X
Qualitative interview			At completion or drop out
Dialysis temperature recording	During each HD session		
Physiological measurements*	During each HD session		
Laboratory measurements**	Measured monthly as part of routine care		
Adverse event reporting	X	X	X
Review Concomitant Medications	X	X	X

*Blood pressure (pre and post HD), intradialytic hypotension, nursing interventions for intradialytic hypotension, intradialytic weight gain over preceding 1 month,
** KT/V as markers of adequate solute clearance, routine haematology and biochemistry
$ Will be monitored every two weeks for the first six weeks
MoCA – Montreal Cognitive Assessment tool, CAM – Confusion Assessment Method, QoL – quality of life, ADL – activities of daily living, HADS – Hospital Anxiety and Depression Scale

Box 1: Questionnaire to assess patient experience of low temperature dialysis

1) Has this low temperuture dialysis session caused you any more discomfort than your usual dialysis session - Yes/No/Not Sure

* If yes, please specify severity of your discomfort

1 2 3 4 5 6 7 8 9 10

(1= minimum, 10= severe)

2) Did you feel cold during dialysis? - Yes/No/Not Sure

3) Did you feel any numbness or pain in your hands and feet? - Yes/No/Not Sure

4) Was your recovery time after dialysis shorter than usual? - Yes/No/Not Sure

5) Do you feel more energetic than your usual dialysis session? - Yes/No/ Not Sure

6) Did you feel sleepier than your usual dialysis? - Yes/No/Not Sure

7) Did you feel more tired than usual dialysis? - Yes/No/Not Sure

8) Did you feel that your concentration was worse than usual? - Yes/No/Not Sure

9) Will you prefer to have low temperature dialysis in the future? - Yes/No/Not Sure

10) If we develop a research project looking at the effect of low temperature dialysis on brain functions; would you be prepared to consider taking part?
- Yes/No/Not Sure
